# Cytoreductive Surgery and Hyperthermic Intraperitoneal Chemotherapy for Gastric Cancer

**DOI:** 10.3390/cancers11111662

**Published:** 2019-10-26

**Authors:** Adriana C. Gamboa, Joshua H. Winer

**Affiliations:** Division of Surgical Oncology, 1365B Clifton Road NE, Suite B4000, Atlanta, GA 30322, USA; jhwiner@emory.edu

**Keywords:** Cytoreductive surgery, intraperitoneal chemotherapy, peritoneal metastases, gastric cancer

## Abstract

The management of peritoneal metastases from gastric cancer origin has evolved considerably over the last three decades with the establishment of cytoreductive surgery (CRS) and hyperthermic intraperitoneal chemotherapy (HIPEC) as efficacious therapies in carefully selected patients. Other approaches such as the use of prophylactic/adjuvant HIPEC in patients who are considered high-risk and those with positive peritoneal cytology will benefit from additional data before being adopted into routine clinical practice. Lastly, there are new and emerging intraperitoneal chemotherapy techniques such as early post-operative intraperitoneal chemotherapy (EPIC) for residual microscopic disease, and pressurized intraperitoneal aerosolized chemotherapy (PIPAC) for patients with advanced unresectable peritoneal carcinomatosis, which are currently under evaluation in clinical trials. The following review outlines the natural history of gastric cancer, currently available neoadjuvant and adjuvant therapies for resectable disease, and existing evidence supporting various approaches to CRS and intraperitoneal chemotherapy.

## 1. Introduction

### 1.1. Epidemiology and Natural History of Gastric Cancer

Gastric cancer is the fifth most common cancer in the United States with an estimated 952,000 incident cases per year [1]. There is widespread geographic variation in its prevalence, with the highest rates occurring in East Asia, South/Central America, and Eastern Europe [2]. Although recent advances and efforts in screening have allowed earlier detection in more endemic areas, most patients are diagnosed at advanced stages, and as such, gastric cancer remains the third leading cause of cancer-related deaths globally [1].

In the United States, approximately 28% of patients present with localized disease for whom the mainstay of treatment remains curative-intent gastrectomy with extended (D2) lymphadenectomy [3]. The remainder of patients will present with local-regional disease or distant spread. Since the publication of the seventh edition of the American Joint Committee on Cancer (AJCC) staging manual, positive peritoneal cytology is classified as M1 disease, even in the absence of visible peritoneal implants, based on previous studies demonstrating that positive cytology is a strong, independent pre-operative predictor of recurrence and survival in patients undergoing R0 resection for gastric cancer [4,5]. The incidence of cytology-positive disease at presentation ranges widely depending on T-stage but has been reported to be as high as 30%, with gross peritoneal carcinomatosis present in almost 20% [6]. Furthermore, even after resection and extended lymphadenectomy, studies of recurrence patterns demonstrate that gastric cancer has the highest rate of peritoneal recurrence of all digestive cancers, with a rate approaching 40%–60% after curative gastrectomy, rising to 80% for those with tumor-positive peritoneal cytology [7,8,9,10,11]. Accordingly, progressive peritoneal carcinomatosis accounts for nearly 60% of deaths from gastric cancer [12].

Historically, the survival rate for gastric carcinoma patients with peritoneal carcinomatosis has been poor, ranging from 2.2 to 8.8 months and no survival at 5 years [13]. Quality and length of life is further worsened by complications such as bowel obstruction, malignant ascites, malnutrition and cachexia, which occur commonly in the setting of peritoneal disease [14,15]. Currently, as peritoneal carcinomatosis is considered a variant of the systemic spread of disease, the standard recommendation for patients with gastric cancer metastatic to the peritoneum is systemic chemotherapy or best supportive care [16]. However, patient-specific novel strategies are being developed to improve the outcome of gastric cancer patients with advanced stage disease. The purpose of this review is to analyze the existing body of literature regarding multimodal treatment strategies for prevention and treatment of peritoneal carcinomatosis from gastric cancer.

### 1.2. Multimodal Treatment of Resectable Gastric Cancer

Although there is no consensus on the optimal treatment approach for resectable gastric cancer, given that local-regional failure is common following curative-intent surgery for gastric cancer and that the pattern of local-regional failure includes both the gastric remnant, the bed of resection, and regional nodal basins, multimodal therapy is paramount and studies have repeatedly validated that combined therapy significantly increases survival in gastric cancer patients with local-regional disease [17,18]. While no universal standard-of-care for the treatment gastric cancer exists, local recommendations are generally followed according to the results of the phase III trials that have been conducted in those areas. Therefore, while perioperative chemotherapy is the preferred treatment strategy for primary gastric cancer in many European countries and the United States, adjuvant chemotherapy is the standard practice in Eastern Asian countries. The therapeutic options for patients with peritoneal metastases, however, are less well-defined, and current treatment recommendations remain controversial.

The following sections highlight current standard-of-care practices for resectable gastric cancer including perioperative and adjuvant systemic chemotherapy, as well as possible therapeutic strategies for the prevention and/or treatment of peritoneal metastases including cytoreductive surgery (CRS) and perioperative chemotherapy which may include neoadjuvant intraperitoneal and systemic chemotherapy (NIPS), hyperthermic intraperitoneal chemotherapy (HIPEC) and/or early post-operative intraperitoneal chemotherapy (EPIC).

### 1.3. Perioperative Chemotherapy for Resectable Gastric Cancer

Peri-operative chemotherapy is the preferred approach for patients with localized, resectable disease in the United States and Europe based on data from two landmark trials in which peri-operative chemotherapy in combination with resection resulted in improved long-term outcomes [19,20]. In the seminal 2006 Medical Research Council Adjuvant Gastric Infusional Chemotherapy (MAGIC) trial, 503 patients with resectable adenocarcinoma of the stomach, esophagogastric junction, or lower esophagus were randomized to surgery with three months of peri-operative chemotherapy with epirubicin, cisplatin and fluorouracil (ECF) or surgery alone. Overall survival (OS) as well as progression-free survival (PFS) were significantly improved in patients who received peri-operative chemotherapy compared with patients treated by surgery alone (*p* = 0.009 and *p* < 0.001, respectively). The 5-year OS rate was 36% for in the peri-operative chemotherapy arm versus 23% for in the surgery only arm. Based on these results, the MAGIC regimen was the preferred peri-operative chemotherapy option for nearly a decade [19]. Recently, the 2019 German phase II/III FLOT-4 trial established the superiority of a peri-operative taxane-based regimen with fluorouracil and leucovorin, oxaliplatin, and docetaxel (FLOT) over peri-operative epirubicin, cisplatin and a fluoropyrimidine or capecitabine (ECF/ECX) in patients with locally advanced, resectable gastric or gastro-esophageal junction adenocarcinoma. The FLOT regimen significantly improved median survival (FLOT: 50 months vs ECF/ECX: 35 months), and led to a higher number of R0 resections (FLOT: 84% vs. ECF/ECX: 77%) [20].

The benefit of neoadjuvant chemotherapy was further established by a Cochrane meta-analysis which reviewed 14 randomized controlled trials investigating the benefit of pre/peri-operative chemotherapy for patients with gastroesophageal adenocarcinoma and found that peri-operative chemotherapy was associated with significantly longer OS (hazard ratio (HR) 0.81, 95% confidence interval (CI) 0.73–0.89, *p* < 0.0001) compared to surgery alone [21]. As these findings have not been replicated in East Asia, neoadjuvant chemotherapy in that region is reserved for patients with locally advanced, marginally resectable gastric cancer, para-aortic and/or bulky nodal disease, and serosa-positive gastric cancer [22].

### 1.4. Adjuvant Chemotherapy and Chemoradiation

In East Asian countries, adjuvant chemotherapy following curative-intent resection without any neoadjuvant therapy is the standard-of-care based on Japanese and Korean trials which showed a clear benefit of adjuvant therapy for stage II or III gastric cancer using S1 (a polypharmaceutic, fluoropyrimidine derivative that combines tegafur with two modulators, gimeracil, and oteracil) administered for one year after surgery or intravenous capecitabine and oxaliplatin (XELOX) [23]. In a 2007 Japanese randomized controlled trial, 529 patients were randomized to D2 gastrectomy followed by S1 beginning within 6 weeks of surgery and continuing for one year, while 530 patients were randomized to D2 gastrectomy alone. The three-year OS was 80% in the S1 group and 70% in the surgery group [24,25]. Subsequently, the capecitabine and oxaliplatin adjuvant study in stomach cancer (CLASSIC) phase III randomized controlled trial undertaken in 37 centers in South Korea, China, and Taiwan randomized 1035 patients to adjuvant chemotherapy with capecitabine plus oxaliplatin or surgery alone. There was a 15% improvement in 3-year disease-free survival (DFS) in the chemotherapy and surgery group (HR 0.56, 95% CI 0.44–0.72, *p* < 0·0001) [26]. A subsequent analysis at 5-year follow-up demonstrated a 9% improvement in OS in the adjuvant capecitabine and oxaliplatin group versus the observation group [27]. Recently, a large 2010 meta-analysis that combined European and Asian data from 17 randomized controlled trials (*n* = 3838) with a median follow-up longer than 7 years, demonstrated an OS benefit of 5.8% at 5 years with post-operative adjuvant fluoropyrimidine-based chemotherapy when compared with surgery alone [28]. Independent European trials of adjuvant chemotherapy have failed to demonstrate similar results and shown no difference between post-operative chemotherapy and surgery alone with D1 lymphadenectomy [29,30,31]. Some of these differences are attributed to marked disparities between the East and the West in both tumor biology—intestinal type and distal stomach location in Asia, versus more diffuse tumors located in the proximal stomach and gastroesophageal junction in the West—and historical surgical practices [32].

The landmark phase II Intergroup-0116 (INT-0116) trial conducted in the United States is the only randomized control trial to support adjuvant chemoradiation for gastric cancer. In this trial, 556 patients with stage IB-IV, M0 gastric cancer were randomized after surgical resection to receive post-operative chemotherapy with 5-FU and leucovorin plus chemoradiation or no additional treatment. After a median follow-up of 5 years, median OS in the surgery-only group was 27 months compared to 36 months in the post-operative chemotherapy plus chemoradiation group (*p* = 0.005) [33]. As only 10% of the patients underwent a D2 lymphadenectomy, the results of the trial are limited as they are only applicable to patients who undergo a D0 or D1 lymph node dissection. This limitation has led to the criticism that chemoradiation was compensating for inadequate surgical clearance of involved lymph nodes thus resulting in improved survival.

The first trial to assess the role of adjuvant chemoradiation after curative-intent gastric cancer resection and D2 lymphadenectomy was the Korean Adjuvant Chemoradiation Therapy in Stomach Cancer (ARTIST) trial where 458 patients with stage IB-IV gastric cancer were randomized to either six cycles of adjuvant capecitabine-cisplatin or to two cycles of capecitabine-cisplatin before and after capecitabine-based chemoradiation. This study demonstrated no difference in 3-year DFS between arms (78.2% vs. 74.2%, *p* = 0.09). Importantly, in the subgroup of patients (*n* = 396) with positive lymph nodes at the time of surgery, patients assigned to the chemotherapy and chemoradiation arm had a slightly improved 3-year DFS when compared to those who received chemotherapy alone (77.5% vs. 72.3%, *p* = 0.04) [34]. Unfortunately, the small sample size within each stage precluded a subgroup stage-specific analysis. Additionally, it is unclear whether the ARTIST data can be applied to Western populations as results from Asian gastric cancer trials have consistently shown improved outcomes compared to Western studies. We currently await the results of the Korean ARTIST-II trial which seeks to compare S1 vs. S1/Oxaliplatin with and without radiotherapy for completely resected gastric adenocarcinoma with D2 lymphadenectomy (NCT01761461).

Several current studies have been designed to further address the optimal sequencing of multimodality therapy in gastric cancer. The Dutch CRITICS trial is comparing pre-operative chemotherapy alone with epirubicin, cisplatin, and capecitabine (ECX) followed by surgery and post-operative ECX alone versus pre-operative ECX followed by surgery and post-operative chemoradiation (NCT00407186) [35]. TOPGEAR is an Australasian, Canadian, and European study evaluating perioperative ECF chemotherapy alone versus perioperative ECF and pre-operative chemoradiation (NCT01924819).

Currently, given the results from the INT-0116 and the ARTIST trials, adjuvant chemoradiation is only recommended for patients who receive less than a D2 lymph node dissection while patients who receive a D2 dissection should be treated with post-operative chemotherapy alone. Unfortunately, chemotherapy and radiotherapy have not shown significant survival advantage as adjuvant treatment for patients with high risk of peritoneal carcinomatosis and locoregional recurrence is still a considerable problem [36,37].

## 2. Approach to Cytoreductive Surgery and Hyperthermic Intraperitoneal Chemotherapy

In patients with peritoneal metastases, systemic chemotherapy alone has had disappointing results. In 1989, Preusser et al. demonstrated that patients with advanced gastric cancer can be treated with an aggressive systemic chemotherapy regimen with a 50% response rate, but was not able to demonstrate a similar response rate in in patients with peritoneal carcinomatosis [38]. Similarly, in 1991, Ajani et al. implemented peri-operative chemotherapy with etoposide, 5-fluorouracil, and cisplatin and found that peritoneal carcinomatosis was the most common reason for treatment failure [39]. Systemic chemotherapy alone is, therefore, not a recommended management plan for patients with peritoneal carcinomatosis. Recently, the use of a multimodality treatment strategy including CRS combined with heated intraperitoneal chemotherapy (HIPEC) has led to promising results in selected patients with peritoneal carcinomatosis of gastric origin. The following section outlines contemporary approaches to CRS and HIPEC in gastric cancer, the currently accepted indications for implementation of this treatment strategy, and other emerging options.

### 2.1. Role of Cytoreductive Surgery and Hyperthermic Intraperitoneal Chemotherapy (HIPEC) in Peritoneal Cancers

For patients at high risk for peritoneal carcinomatosis from gastric cancer, treatment remains limited as neither neoadjuvant nor adjuvant treatment approaches have been shown to decrease the progression to peritoneal carcinomatosis. Importantly systemic therapies have limited effects on peritoneal carcinomatosis likely due to the blood–peritoneal barrier consisting of stromal tissue between mesothelial cells and sub-mesothelial blood capillaries [12]. The resulting median survival for these patients is, therefore, as low as 5 months [40].

Multimodal therapeutic approaches combining CRS and peritonectomy with peri-operative intraperitoneal chemotherapy have emerged over the last four decades after this approach was first proposed in 1980 by Spratt in canine models, with the rationale being that it treats the most common pattern of failure that occurs after gastric cancer surgery [41]. The technique of CRS followed by HIPEC was further advanced by Sugarbaker in the 1990s [42]. The foundation of both of these practices can be explained by Gompertzian cellular kinetics which postulate that in the initial stages, tumor cell growth is exponential. As the tumor enlarges, its blood supply diminishes thus slowing down tumor growth. Tumor debulking thereby allows the cells to re-enter the proliferative phase of the cell cycle, potentially becoming more sensitive to anti-neoplastic agents. The importance of debulking by CRS is further underscored by the concept that intraperitoneal chemotherapy penetrates the peritoneal nodule by simple diffusion and has limited penetration of only 2–3 mm of tissue [36,43,44]. Hyperthermia between 39 °C and 43 °C additionally enhances the chemosensitivity of tumor cells to the cytotoxic agents and increases the effectiveness of certain agents (mitomycin C, cisplatin, oxaliplatin), while achieving drug concentrations that are up to 20 times higher compared to plasma levels and minimizing systemic toxicity due to the blood peritoneal barrier [13,45].

The importance of patient selection for CRS and intraperitoneal chemotherapy cannot be overstated as a large number of patients with gastric peritoneal metastases will not benefit from CRS and HIPEC due to the extent of disease. Accurate pre-operative imaging not only assists in planning cytoreduction, but also prevents an unwarranted laparotomy in patients who have unresectable disease. The extent of peritoneal metastases as measured by Sugarbaker’s peritoneal cancer index (PCI) significantly influences completeness of cytoreduction and subsequent survival [46]. The current literature for the use of HIPEC for peritoneal metastases of gastric cancer origin has significant variation related to treatment intent, stage of disease, surgical technique, and intraperitoneal chemotherapy agent utilized. The following sections outline some of the important advances that have been made over the past four decades in each of these areas for the treatment of gastric cancer.

### 2.2. Drugs Used in HIPEC

Multiple drugs have been used in HIPEC for gastric cancer and there is currently no consensus regarding the optimal drug regimen or dosing strategy. Mitomycin C, cisplatin and taxanes are the most commonly used agents as they display the characteristics of an ideal drug including proven systemic activity, synergistic activity with hyperthermia, and concentration-related toxicity. Mitomycin C is an alkylating tumor antibiotic and was the first drug used as monotherapy for HIPEC. It is usually given in a dose of 15 mg/m^2^ for 90 minutes [7,47]. The most common regimen consists of 40 mg mitomycin C for 90–120 minutes. Platinum-based alkylating agents such as cisplatin and oxaliplatin are commonly employed, typically in combination with mitomycin C [48]. Cisplatin has been given in doses ranging from 50 to 200 mg/m^2^ with perfusion time between 60 and 90 minutes [49]. Oxaliplatin is usually dosed at 460 mg/m^2^ for either 30 or 60 minutes as it is rapidly taken up by tumor tissue [50]. These two agents are also generally administered with a metal binding agent such as sodium thiosulfate to prevent renal toxicity. Additionally, due to synergistic effect of oxaliplatin with 5-FU, oxaliplatin can be administered with concurrent intravenous 5-FU and leucovorin as part of bidirectional therapy either just prior to, or during, hyperthermic intraperitoneal chemotherapy [51].

### 2.3. Intraperitoneal Chemotherapy as Neoadjuvant Approach

The first study to consider the use of HIPEC as a neoadjuvant approach prior to gastrectomy in patients with positive peritoneal cytology or low-volume peritoneal carcinomatosis was published in 2017 by Badgwell et al. In this single-arm phase II trial, 19 patients received laparoscopic HIPEC with a combination of mitomycin C 30 mg and cisplatin 200 mg. Approximately half of the patients (48%) received two to five laparoscopic procedures. After the final HIPEC, seven patients had negative peritoneal cytology and no peritoneal carcinomatosis, and five of these patients underwent definitive surgery. For these five patients, the median OS from date of resection was 29 months while the median OS from the date of diagnosis of metastatic disease for the whole group was 30.2 months. Although this was limited by a small sample size, results demonstrate the feasibility of integrating neoadjuvant HIPEC into the therapeutic armamentarium for gastric cancer [52]. Additionally, this study established laparoscopy as a potential tool for evaluating extent of peritoneal disease below the threshold of imaging modalities [53]. This same group further demonstrated the safety of laparoscopy for gastric HIPEC in a 2019 retrospective study of 77 laparoscopic HIPEC procedures in 44 patients with peritoneal carcinomatosis or peritoneal positive cytology. In this study, there were no conversions to laparotomy and median length of stay was two days. Importantly, 25% of patients underwent secondary gastrectomy following resolution of positive peritoneal cytology [54].

In 2006, a new bidirectional chemotherapy therapeutic strategy for patients with peritoneal carcinomatosis from gastric cancer was proposed which includes neoadjuvant intraperitoneal and systemic chemotherapy (NIPS) [55]. The rationale for this technique is to target peritoneal carcinomatosis via the systemic circulation and by diffusion from within the peritoneal cavity to enable a complete cytoreduction. In the 2006 study by Yonemura et al., patients with histologically or cytologically proven peritoneal carcinomatosis from gastric adenocarcinoma underwent placement of a peritoneal port system which allowed infusion of 400 mg of docetaxel and 150 mg of carboplatin with simultaneous intravenous infusion of methotrexate and 5-FU. A minimum of two cycles and up to six cycles of NIPS were used prior to cytoreduction. Treatment resulted in negative peritoneal cytology in 56% of patients and those who received a complete resection had a median survival of 20.4 months compared to 14.4 months in all patients [55]. In a subsequent 2012 study by Yonemura et al., 96 patients with histologically or cytologically proven peritoneal carcinomatosis underwent similar treatment with intraperitoneal Taxotere and cisplatin with infusions on days 1, 8, and 15 in conjunction with oral S1 for 21 days. This cycle was performed twice prior to surgery. Among all the enrolled patients, 82 were eligible for CRS, gastrectomy, peritonectomy and D2 lymphadenectomy. Complete pathologic response was achieved in 36.8%, and complete cytoreduction (CC-0) in 70%. Importantly, grade 3 and 4 complications were reported in 10%, which is approximately the same as reported for CRS and HIPEC in the absence of NIPS. Median survival of patients who were able to undergo CRS was 14.4 months compared to 9.0 months in patients who were not able to undergo CRS (*p* = 0.032) [12].

In 2014, Canbay et al. assessed early- and long-term outcomes of NIPS in 194 patients with positive peritoneal cytology. After induction treatment, 78% of patients who showed negative cytology underwent CRS and HIPEC. Similar to the 2012 study by Yonemura et al., complete response was achieved in 24%, and complete cytoreduction was achieved in 68%. For those who underwent definitive surgery, median survival was 15.8 months compared to 7.5 months for patients who did not undergo surgery due to persistent positive cytology or peritoneal deposits (*p* < 0.001). Complete cytoreduction resulted in a significantly higher median survival of 20.5 months compared to 10.9 months for those who underwent attempted debulking. Post-operative complications occurred in 24%, and post-operative mortality rate was 4%. Multivariable analysis identified pathologic response, low tumor burden (PCI ≤6), and completeness of cytoreduction (CC-0/CC-1; all *p* = 0.001) as independent predictors for a better prognosis. Notably, this study also showed that 78 patients with ascites had improvement in their symptoms thus further establishing the role of NIPS as a palliative technique [56]. As evidenced here, there are currently several regimens used for NIPS but none have been compared in clinical trials.

Based on these data, NIPS should be considered in gastric cancer patients with low-volume peritoneal metastases (PCI ≤6) or symptomatic ascites. Clinical trials are needed to further establish this approach to CRS and HIPEC and none have occurred to date.

### 2.4. HIPEC as Prophylactic/Adjuvant Approach

Given the negative prognostic value of positive peritoneal cytology and peritoneal carcinomatosis in gastric cancer, it is imperative to prevent peritoneal recurrence after radical surgery in patients at high-risk. One of the most recent therapeutic approaches for patients who are considered high-risk for peritoneal recurrence is the administration of intraperitoneal adjuvant chemotherapy soon after curative-intent resection. The benefit of using HIPEC as an adjuvant treatment for locally advanced but resectable gastric cancer without pre-operatively confirmed peritoneal disease has been reported in several phase II clinical trials from Japan and China and a single meta-analysis.

In 1988, Koga et al. were the first to report the long-term benefit in survival of adding HIPEC to curative gastrectomy (3-year survival of 74% with HIPEC and 53% without HIPEC, *p* < 0.04) [47]. In 2001, Yonemura et al. randomized 139 patients with intraoperative findings of serosal involvement to surgery and HIPEC with mitomycin and cisplatin, surgery and intraperitoneal chemotherapy without hyperthermia, or surgery alone. Overall 5-year survival rate was 61% in the HIPEC group as opposed to 43% and 42% in the other two groups [50]. Also in 2001, Kim et al. performed a prospective controlled study for 52 patients who were selected to undergo surgery and HIPEC with mitomycin C versus 51 patients who underwent surgery alone. 5-year survival rate was significantly higher in the surgery and HIPEC group (59%) compared to surgery alone (44%) when stage IV (peritoneal carcinomatosis) patients were excluded (*p* = 0.04) [57].

The remaining studies which have sought to address the role of HIPEC as a prophylactic/adjuvant therapeutic approach are summarized in Table 1. Recently, a few systematic reviews and meta-analyses have also sought to understand the benefit of adjuvant HIPEC. A 2007 meta-analysis by Yan et al. included 13 studies in which patients with locally advanced gastric cancer were randomized to receive surgery combined with intraperitoneal chemotherapy versus surgery alone. There was a survival benefit associated with HIPEC (HR 0.60, 95% CI 0.43–0.83, *p* = 0.002) or HIPEC with EPIC (HR 0.45, 95% CI 0.29–0.68, *p* = 0.0002) as well as a small benefit with normothermic intraoperative intraperitoneal chemotherapy, but no significant improvement in survival with EPIC alone or delayed post-operative intraperitoneal chemotherapy [58]. Similarly, Coccolini et al. performed a systematic review in 2014 to evaluate the effects of intraperitoneal chemotherapy in patients with advanced gastric cancer with or without peritoneal carcinomatosis. Results demonstrated that 1-, 2- and 3-year OS was improved by the intraperitoneal chemotherapy in all included patients and in patients with locoregional nodal metastasis or serosal infiltration [59]. A recent 2018 meta-analysis by Desiderio et al. included 11 randomized controlled trials and 21 studies comprising a total of 2520 patients comparing surgery with HIPEC and standard surgical management for the treatment of advanced stage gastric cancer with and without peritoneal carcinomatosis. Analysis demonstrated a significant 3-year and 5-year survival benefit to HIPEC compared to surgery alone (3-year: risk ratio [RR] = 0.71, 95% CI 0.53–0.96, *p* = 0.03; 5-year: RR = 0.82, 95% CI 0.70–0.96, *p* = 0.01) [60]. Despite the inclusion of some patients with peritoneal involvement in these studies and the heterogeneity with respect to the drugs used, results demonstrate that long-term outcomes can be improved with intraoperative peritoneal chemotherapy in the adjuvant/prophylactic setting. Ultimately, these analyses cannot be used to determine the best regimen of intraperitoneal chemotherapy for advanced gastric cancer. Additionally, several questions remain to be answered including the optimal choice of agent, dosage and duration of treatment.

### 2.5. HIPEC with Curative Intent for Cytology Positive Peritoneal Carcinomatosis

To date, no trials have been conducted to evaluate the effect of HIPEC on cytology-only positive peritoneal carcinomatosis. A prospective phase III multi-institutional randomized controlled trial is currently ongoing in France to compare 5-year OS of patients with gastric cancer involving the serosa and/or lymph node involvement and/or with positive cytology at peritoneal washing who undergo curative gastrectomy and D2 resection with and without HIPEC with Oxaliplatin. Secondary end points include DFS, morbidity, pattern of recurrence, and quality of life (GASTRICHIP, NCT01882933) [9].

### 2.6. HIPEC with Curative Intent for Macroscopic Peritoneal Carcinomatosis

The majority of the literature regarding the benefit of CRS and HIPEC in gastric cancer has included patients with macroscopic peritoneal disease (Table 2). Some of these studies, while initially designed to evaluate HIPEC in the adjuvant/prophylactic setting in patients undergoing a potentially curative resection, include separate analyses of patients that were unexpectedly found to be stage IV at operation but still underwent resection of serosal deposits followed by HIPEC. So far, however, only a few randomized clinical trials have been conducted to support such a treatment strategy. The initial trials conducted in 1983 through 1993 included few patients with stage IV disease [7,50,61]. A 2007 meta-analysis summarized results from these early trials and found a significant survival improvement in favor of surgery and HIPEC compared with surgery alone (HR 0.60, 95% CI 0.43–083, *p* = 0.002) [58].

In 2004, Glehen et al. conducted a prospective trial with 49 patients with peritoneal carcinomatosis who underwent HIPEC with 40–60 mg of mitomycin C. The overall 1-, 2- and 5-year survival rates were 48%, 20%, and 16%, respectively. Significant independent predictors of worse survival were pre-operative malignant ascites and CCR-2 (diameter of residual nodules >5 mm) [69]. A more recent 2011 prospective randomized phase III trial by Yang et al. compared CRS alone versus CRS and HIPEC with mitomycin C and cisplatin for peritoneal carcinomatosis secondary to gastric cancer in patients with both similar PCI and CCR scores. Results demonstrated that CRS and HIPEC significantly improved survival by nearly 70% (6.5 vs. 11.0 months) compared with CRS alone (*p* = 0.046) [61]. Subsequently, the prospective CYTO-CHIP study in France confirmed the benefit of HIPEC in gastric peritoneal carcinomatosis in the Western population with a 5-year OS of 19.9% for patients treated with CRS and HIPEC (*n* = 180) versus 6.4% for those treated with CRS alone (*n* = 97) [73]. As large prospective randomized trials are lacking in Western patients, the GASTRICHIP trial is being undertaken in France. Over 300 patients will be randomized intraoperatively to receive gastric resection with or without HIPEC with Oxaliplatin, with a primary outcome of 5-year OS. Quality of life outcomes will also be evaluated [9].

Several studies have demonstrated that when PCI is higher than 12, survival remains poor, even with a complete cytoreduction. Indeed, in 1996 Fujimoto et al. reported a 5-year survival of 40–50% for patients with limited peritoneal metastases, but a 1-year survival of only 18% for patients with numerous distant peritoneal metastases, according to the classification system by the Japanese Research Society for Gastric Cancer [68,74]. More recently, Glehen et al. reported that PCI is the most important prognostic factor in patients who are able to undergo complete CRS. The results of their randomized controlled trial demonstrated that no patient was alive at 6 months if they had a PCI greater than 19 and none at 3 years if they had a PCI greater than 12 [37]. Therefore, CRS and HIPEC should be carefully considered for patients with a PCI of 12 of lower to ensure a therapeutic benefit. Lastly, given the associated morbidity of CRS, patient selection is paramount and should take into account pre-operative functional status.

### 2.7. Early Post-Operative Intraperitoneal Chemotherapy (EPIC)

Early post-operative intraperitoneal chemotherapy has been proposed as a strategy to eliminate residual microscopic peritoneal disease after resection of stage III gastric cancer. EPIC regimens comprising either mitomycin C and 5-FU or taxanes are usually administered on post-operative days 1–5 through inflow and outflow catheters which are inserted at the time of CRS with repeated instillations every 24 hours. EPIC is administered early in the post-operative period prior to adhesion formation to optimize uniform distribution of the intraperitoneal agents [75]. Three studies have evaluated the use of EPIC after resection of gastric cancer.

Yu et al. compared curative gastric resection with or without EPIC with mitomycin C and 5-FU. The addition of EPIC after gastric resection resulted in improved 5-year OS compared to surgery alone (54% vs. 38%). In patients with stage III and stage IV disease, there was a significant increase of 5-year OS (stage III: 57% vs. 23%, *p* = 0.0098 and stage IV: 28% and 5%, *p* = 0.0098) in the EPIC group [76]. In a retrospective study by Kwon et al., 245 patients with stage III, serosa-invading, gastric cancer underwent either curative resection alone (*n* = 180) or curative resection and EPIC (*n* = 65). The 5-year OS was 47.4% in the EPIC group and 26.7% in the non-EPIC group (*p* = 0.012) while the 5-year DSS rates were 53.1% in the EPIC group compared to 29.7% in the non-EPIC group (*p* = 0.011). Peritoneal recurrence rates for the EPIC group and the non-EPIC group were 18.5% and 32.2%, respectively (*p* = 0.038) [77]. EPIC is being further evaluated in the Korean EPIC-GC trial (NCT02205008) where patients with gastric adenocarcinoma who are candidates for curative D2 resection of the stomach are randomized to either EPIC (mitomycin C and 5-FU) and adjuvant systemic chemotherapy employing S1 or adjuvant systemic chemotherapy with S1 alone. The primary end point is 3-year PFS. We currently await results from this trial which finalized accrual in late 2018. In the meantime, the addition of EPIC to CRS/HIPEC can be considered in patients with good performance status who are able to undergo a complete cytoreduction with no prior extensive pre-treatment.

### 2.8. HIPEC as Palliative Approach

One of the most frequent and debilitating complications of peritoneal carcinomatosis is the accumulation of malignant ascites which can result in a multitude of symptoms including abdominal pain, shortness of breath, early satiety in addition to fatigue, depression and anxiety [78]. The etiology of malignant ascites is complex and caused by the combined effects of tumor-produced proteins and impaired lymphatic drainage [79]. Although frequent paracentesis can help palliate symptoms, the effects only last approximately 72 hours, thus providing limited improvement to a patient’s quality of life [80]. HIPEC has, therefore, been employed to decrease re-accumulation of ascites. Some of the first studies to report successful results were performed by Fujimoto et al. and Yonemura et al. with resolution of ascites in nearly 78% of patients [81,82]. Recently, laparoscopic HIPEC has also shown promising results for the treatment of malignant ascites as it reduces operative time and hospital length of stay. In a systematic review involving 76 patients who underwent palliative laparoscopic HIPEC, ascites control was achieved in 95% patients with no major complications and a 7.6% incidence of minor complications [82].

Although palliative CRS addresses the underlying tumor burden, morbidity is high and recovery is long thus limiting its indications. A 2014 retrospective review of 299 prospectively maintained CRS/HIPEC procedures performed for patients with ascites from various primary tumors (gastric adenocarcinoma: 6%) found that CRS/HIPEC is 93% effective in successfully controlling ascites at three-month follow-up even when a complete cytoreduction is not achieved [83]. Although not explicitly published in this study, the resolution of ascites has been found to persist until death due to progressive disease [84]. These data suggest that palliative CRS/HIPEC may be effective in gastric cancer. However, more robust studies are needed to establish this technique in routine clinical practice.

## 3. Future Areas of Study

The data presented here provide a foundation on which to develop more prospective trials, particular in Western populations, to justify the inclusion of CRS and HIPEC in national treatment guidelines. Many questions remain to be addressed including the optimal intraperitoneal chemotherapy agent and dosing, the optimal timing and sequence of intraperitoneal chemotherapy, strategies to further reduce morbidity and the patient population who will most benefit from these interventions. Currently, various trials are ongoing in Europe and China using varying doses and dwelling time of oxaliplatin, oxaliplatin and paclitaxel, and mitomycin and cisplatin for peritoneal carcinomatosis or prophylactic HIPEC for locally advanced gastric cancer (Table 3).

As the research to answer these questions continues, new intraperitoneal techniques are being developed to help address some of the shortcomings of HIPEC and EPIC and expand the patient population eligible for these procedures. Pressurized intraperitoneal chemotherapy (PIPAC) is a new technique that uses aerosolized chemotherapy to produce a high tumor drug concentration and deeper penetration. During PIPAC, laparoscopic access is obtained to create a pneumoperitoneum of 12 mm Hg and nebulized chemotherapy is given to create capnoperitoneum which is maintained for 30 minutes [85]. The increase in intra-abdominal pressure is thought to aid tissue uptake and intra-tumoral drug concentration [86]. An ongoing German trial (PIPAC GA-01; NCT01854255) is studying the clinical benefits of PIPAC (cisplatin and doxorubicin) in patients with recurrent gastric cancer. PIPAC is currently used for symptom palliation, but more evidence is needed before its indications can be expanded.

One of the challenges of intraperitoneal therapy is the delivery of sufficiently high intraperitoneal drug concentrations to provide a large concentration gradient between the peritoneal cavity and tumor tissue. Several drug-delivery systems such as nanoparticles, microspheres and hydrogels, are currently being developed and tested in order to maximize peritoneal concentration while continuing to minimize systemic toxicity [87]. Various immunotherapies are also being evaluated for intraperitoneal use including immune checkpoint inhibitors, chimeric antigen receptor-T cells (CAR-T Cells), and bevacizumab [88,89,90]. Catumaxomab, a rat-mouse hybrid monoclonal antibody, is a new agent that is being evaluated in phase II/III trials for use in patients with malignant ascites from gastric cancer after two recent studies demonstrated promising results [91,92]. One study showed an acceptable safety profile with minimal adverse events including fever, vomiting, and abdominal pain [91]. In the second phase II/III trial, patients with recurrent symptomatic malignant ascites were randomized to paracentesis plus intraperitoneal catumaxomab or paracentesis alone. Puncture-free survival was significantly longer in the catumaxomab group compared to the control group (median 46 vs. 11 days, HR: 0.254, *p* < 0.0001). Additionally, catumaxomab patients had fewer signs and symptoms of ascites than control patients [92]. Selecting the patients who will benefit most from these therapies will largely depend on the development of personalized approaches as we continue to identify ways to detect molecular alterations in tumor tissue via next-generation sequencing. Cell-free circulating tumor DNA in patients with peritoneal metastases has the potential to help disease prognostication and further identify optimal therapeutic strategies [93].

## 4. Conclusions

Current studies demonstrate that there is an emerging role for the use of prophylactic HIPEC to prevent the incidence of peritoneal metastases for high-risk patients with gastric cancer. Identification of patients with gastric cancer at high risk of developing peritoneal metastasis, standardization of the drugs and their dosage, and robust results from ongoing trials will ensure its inclusion in the treatment armamentarium for resectable gastric cancer.

For patients with established peritoneal carcinomatosis, current approaches for intraperitoneal chemotherapy include the use of NIPS, as curative-intent in both cytology-positive and macroscopic disease, and as EPIC in the setting of microscopic residual disease. The efficacy of these approaches relies on the possibility of achieving complete cytoreduction due to the limited drug action on a residual tumor nodules larger than 2.5 mm (CC0-1) [94]. Confirmation of burden of disease as measured by the PCI should be obtained with high-quality cross-sectional imaging, although diagnostic laparoscopy can also be used as a tool to evaluate the extent of peritoneal disease. Given study findings that demonstrate a benefit in survival, CRS/HIPEC is currently the optimal treatment in carefully selected patients with peritoneal carcinomatosis from gastric cancer origin with a PCI <12. More studies are needed to further establish EPIC as a viable therapeutic strategy as the clinical data reporting the benefit of EPIC for gastric cancer is lacking and its current role remains undefined. Further research on the use of EPIC for gastric cancer should focus on the duration of drug instillation and number of cycles needed to result in improved long-term outcomes. There is still very limited data on the use of HIPEC as palliative approach for malignant ascites, though some studies suggest that it may be effective in gastric cancer. As such, the use of this technique is currently not established in clinical practice. Lastly, the therapeutic landscape for the treatment of peritoneal carcinomatosis continues to evolve with the development of new drugs and delivery systems as well as new techniques, such as PIPAC, which are actively being evaluated in clinical trials. Progress in the field of CRS and intraperitoneal chemotherapy will depend of effective collaboration between clinicians of various specialties and between institutions worldwide. Currently, there is a lack of large-scale, multi-institutional phase III trials to further assess the value of HIPEC in patients with gastric cancer likely because of the difficulty of performing these trials due to variability in surgeons’ styles, perioperative care, and lack of standardization for the delivery of intraperitoneal chemotherapy. Although accrual of sufficient patients in these trials remains a challenge, the recently reported results from the PRODIGE-7 trial in colorectal cancer confirm that large-scale prospective trials in the field of HIPEC are feasible [95].

## Figures and Tables

**Table 1:** Prospective or randomized controlled studies evaluating the effect of hyperthermic intraperitoneal chemotherapy (HIPEC) as prophylactic/adjuvant approach.

Author	Year	Country	Number of Patients	Agent	Outcome
Koga [47]	1988	Japan	25 (surgery + HIPEC) vs. 21 (surgery only)	MMC 64–100 mg	30-month OS: 83% vs. 67%, *p* = 0.001
Hamazoe [61]	1994	Japan	41 (surgery + HIPEC) vs. 39 (surgery alone)	MMC 10 μg/mL	Median OS: 77 vs. 66 months 5-year OS: 64% vs. 52%
Ikeguchi [62]	1995	Japan	75 (surgery + HIPEC) vs. 96 (surgery alone)	MMC 80–100 mg/m^2^	5-year OS: 51% vs. 46%
Fujimoto [7]	1999	Japan	69 (surgery + HIPEC) vs. 68 (surgery alone)	MMC 10 mg/mL	2, 4, 8-yr OS (88% vs. 77%, 76% vs. 58%, 62% vs. 49%)
Hirose [63]	1999	Japan	15 (surgery + HIPEC) vs. 39 (surgery alone)	MMC 20 mg, Cisplatin 100 mg, VP16 100 mg	Median OS: 33 vs. 22 months 3, 5-yr OS (49% vs 29%, 39% vs. 17%)
Kim [57]	2001	Korea	51 (surgery + HIPEC) vs. 50 (surgery alone)	MMC 40 mg	5-yr OS: 32.7% vs. 27.1%
Zhu [64]	2006	China	41 (surgery + HIPEC) vs. 53 (surgery alone)	MMC 30 mg, Cisplatin 300 mg	2, 4, 6-yr OS (83% vs 64%, 71% vs 52%, 68% vs 38%)

HIPEC—hyperthermic intraperitoneal chemotherapy, MMC—mitomycin C.

**Table 2:** Prospective or Randomized controlled studies evaluating the effect of HIPEC in established peritoneal carcinomatosis.

Author	Year	Country	Number of Patients	Agent	Duration (min)	Outcome
Fujimoto [65]	1990	Japan	20 (CRS + HIPEC) vs. 7 (CRS only)	MMC 10 μg/mL	120	6-mo survival: 94% vs. 57%, *p* = 0.001
Yonemura [66]	1991	Japan	41	MMC 5 μg/mL Cisplatin 30 μg/mL	40–60	Median survival: 14.5 mo 3-yr survival: 28.5%
Yonemura [67]	1996	Japan	83 (surgery + HIPEC)	MMC 30 mg Cisplatin 300 mg Etoposide 150 mg	60	5-yr survival (overall: 11%, CCR0/1: 17%, CCR2: 2%)
Fujimoto [68]	1997	Japan	48 (CRS + HIPEC) vs. 18 (CRS only)	MMC 10 μg/mL	120	1, 3, 5, 8-yr survival (CRS + HIPEC vs. CRS: 54% vs. 11%, 42% vs. 0%, 31% vs. 0%, 25% vs. 0%; *p* = 0.001)
Glehen [69]	2004	France	49 (CRS + HIPEC)	MMC 40–60 mg	90	Median survival (overall: 10.3 mo; CCR0/1 vs. CCR2: 21.3 vs. 6.6 mo, *p* < 0.001)
Yang [70]	2010	China	28 (CRS + HIPEC)	MMC 30 mg Cisplatin 120 mg	90–120	2-yr survival: 43% Median survival (PCI ≤ 20 vs PCI > 20): 27.7 vs. 6.4 mo, *p* = 0.0001
Yang [71]	2011	China	34 (CRS + HIPEC) vs. 34 (CRS only)	MMC 30 mg Cisplatin 120 mg	60–90	Median survival (CRS + HIPEC vs. CRS): 12 vs. 6.5 mo, *p* = 0.02
Magge [72]	2014	USA	23 (CRS + HIPEC)	MMC 40 mg	100	Median survival: 9.5 mo 3-yr survival: 18%

CRS—cytoreductive surgery, HIPEC—hyperthermic intraperitoneal chemotherapy, MMC—mitomycin C.

**Table 3:** Active gastric cancer CRS and HIPEC clinical trials.

Trial	Country	*n*	Treatment	Agent	Primary Endpoint	Estimated Completion
NCT03092518	USA	40	CRS + HIPEC	Cisplatin + mitomycin C + sodium thiosulfate	Overall survival	October 2020
NCT02356276	China	584	CRS + post-operative HIPEC + systemic chemotherapy vs. CRS alone + systemic chemotherapy	Paclitaxel	Overall survival	January 2022
NCT02891447	USA	30	CRS + HIPEC	Mitomycin C + Cisplatin	Overall survival	September 2021
NCT02158988	Germany	180	Neoadjuvant chemotherapy + CRS + HIPEC vs. neoadjuvant chemotherapy + CRS alone	Mitomycin C + Cisplatin	Overall survival	September 2020
NCT02960061	China	640	Neoadjuvant chemotherapy + CRS + HIPEC + adjuvant chemotherapy vs. neoadjuvant chemotherapy + CRS + adjuvant chemotherapy	Paclitaxel	Overall survival	December 2019
NCT03023436	China	220	CRS + HIPEC + systemic chemotherapy vs. systemic chemotherapy alone	Cisplatin + Fluoropyrimidine	Median survival	June 2022
NCT02969122	China	59	Neoadjuvant HIPEC + neoadjuvant chemotherapy ± CRS vs. CRS + HIPEC + adjuvant chemotherapy	Docetaxel	Overall survival	December 2023
NCT02381847	China	60	CRS + HIPEC vs. CRS alone + adjuvant chemotherapy	Cisplatin	Overall survival	March 2020
NCT01882933	France	322	CRS + HIPEC vs. CRS alone	Oxaliplatin	Overall survival	May 2025
